# Genetic Variants in Transcription Factor Binding Sites in Humans: Triggered by Natural Selection and Triggers of Diseases

**DOI:** 10.3390/ijms22084187

**Published:** 2021-04-18

**Authors:** Chia-Chun Tseng, Man-Chun Wong, Wei-Ting Liao, Chung-Jen Chen, Su-Chen Lee, Jeng-Hsien Yen, Shun-Jen Chang

**Affiliations:** 1 Graduate Institute of Clinical Medicine, College of Medicine, Kaohsiung Medical University, Kaohsiung 80708, Taiwan; 990331kmuh@gmail.com (C.-C.T.); jehsye@kmu.edu.tw (J.-H.Y.); 2 Division of Rheumatology, Department of Internal Medicine, Kaohsiung Medical University Hospital, Kaohsiung 80756, Taiwan; 3 Department of Biotechnology, College of Life Science, Kaohsiung Medical University, Kaohsiung 80708, Taiwan; harry556123@gmail.com; 4 Department of Medical Research, Kaohsiung Medical University Hospital, Kaohsiung 80756, Taiwan; 5 Department of Internal Medicine, Kaohsiung Municipal Ta-Tung Hospital, Kaohsiung 80145, Taiwan; chungjencgmh@gmail.com; 6 Laboratory Diagnosis of Medicine, College of Medicine, Kaohsiung Medical University, Kaohsiung 80708, Taiwan; sclee@kmu.edu.tw; 7 Institute of Biomedical Sciences, National Sun Yat-Sen University, Kaohsiung 80424, Taiwan; 8 Department of Biological Science and Technology, National Chiao-Tung University, Hsinchu 30010, Taiwan; 9 Department of Kinesiology, Health and Leisure Studies, National University of Kaohsiung, Kaohsiung 81148, Taiwan

**Keywords:** gout, transcription factor binding sites, natural selection, histone modification, methylation, chromatin conformation

## Abstract

Variants of transcription factor binding sites (TFBSs) constitute an important part of the human genome. Current evidence demonstrates close links between nucleotides within TFBSs and gene expression. There are multiple pathways through which genomic sequences located in TFBSs regulate gene expression, and recent genome-wide association studies have shown the biological significance of TFBS variation in human phenotypes. However, numerous challenges remain in the study of TFBS polymorphisms. This article aims to cover the current state of understanding as regards the genomic features of TFBSs and TFBS variants; the mechanisms through which TFBS variants regulate gene expression; the approaches to studying the effects of nucleotide changes that create or disrupt TFBSs; the challenges faced in studies of TFBS sequence variations; the effects of natural selection on collections of TFBSs; in addition to the insights gained from the study of TFBS alleles related to gout, its associated comorbidities (increased body mass index, chronic kidney disease, diabetes, dyslipidemia, coronary artery disease, ischemic heart disease, hypertension, hyperuricemia, osteoporosis, and prostate cancer), and the treatment responses of patients.

## 1. Introduction

For the past decade, genome-wide association studies (GWASs) have advanced the knowledge of populations and complex trait genetics, understanding of the biology of diseases, and the clinical translation of new therapeutics. A standard GWAS involves investigators comparing common genetic variants found in the genomes of affected cases with the sequences in a control group to determine whether an association exists. The analysis of genomic variation by GWAS provides unprecedented opportunities for understanding the pathophysiology of complex traits, including susceptibility to a particular disease. An important insight emerging from GWAS, is that the vast majority of significant genetic variants are located in noncoding regions. For example, of 465 unique trait/disease-associated single nucleotide polymorphisms derived from 151 GWASs, only 12% are located in protein-coding regions, while 45% fall within introns and another 43% fall within intergenic regions [1]. Although most GWAS-identified polymorphisms are located in noncoding regions, according to a past review, the majority of well-studied polymorphisms are within protein-coding regions because there is an absence of functional annotation for noncoding variants [2,3]. Given that most trait-associated variants are located in noncoding regions, these causal variants and traits do not appear to be linked with the eventual amino acid sequences and accompanying protein functions, such as DNA binding, catalytic activity, and ligand–receptor interaction. A plausible effect of the trait-associated variants in noncoding regions would be differential gene expression.

Since transcription factors recognize and bind specific DNA sequences in areas called binding sites and affect the expression of target genes [4], one major explanation for why causal variants of transcription factor binding sites (TFBSs) alter gene expression could be that the causal variants perturb transcription factor binding, and therefore, alter gene expression [5]. TFBSs may be located in close proximity to or even within the genes they regulate. However, they can also be found at considerable distances from the genes [6]. Transcription factors act as molecular switches to regulate the amount and timing of gene transcription [6]. Therefore, the sequence-specific binding of transcription factors to the regulatory regions in the DNA is proposed to be a key regulatory mechanism that determines gene expression and, hence, heritable phenotypic variation and the onset and/or severity of disease [6,7]. Based on this concept, it could be anticipated that the regulatory elements of TFBSs contribute to genetic elements in disease. Previous analyses provide solid evidence supporting such an argument:

(1) TFBS polymorphisms comprise only 8% of the genome polymorphisms but 31% of the trait-associated polymorphisms identified by GWAS [8].
(2) Up to 21.6% of variants affecting gene expression overlap annotated TFBSs [9].
(3) Polymorphisms leading to the differential binding of transcription factors are highly enriched in the set of causal variants reported for traits across several independent studies [10].
(4) Chromatin immunoprecipitation followed by high-throughput sequencing (ChIP-seq) has demonstrated the extensive contributions of genetic variations to transcription factor binding and a significant correlation between nucleotide changes affecting transcription factor binding and gene expression [11].

Taken together, the findings presented above suggest a strong role for TFBS variation in downstream gene expression and phenotypic variation.

## 2. Genomic Features of TFBSs and Genetic Variants of TFBSs

TFBSs make up only a small proportion of human DNA sequences [12]. TFBSs are associated with various genomic features. Enhancers contain more than 60% of the identified TFBSs, ~20% of TFBSs are located near transcription start sites, and more than 10% of TFBSs are found in promoter-proximal regions [13]. The probability of a sequence being a TFBS differs with respect to the DNA CpG content. Past studies have shown that most transcription factors bind to promoters with high CpG contents [12]. Generally, different transcription factors show distinct distributions across the genome, with some transcription factors concentrated in the transcription start site, while other sets of transcription factors are enriched in enhancers [12].

There are also some epigenetic signatures associated with TFBSs. A total of 98.5% of the occupancy sites of transcription factors mapped by ENCODE ChIP-seq lie within accessible chromatin, defined by DNaseI hotspots [12]. The average H3K27me3, H3K36me3, and H4K20me1 modification levels are reduced by 21%, 9.6%, and 17%, respectively, in TFBS regions compared to non-TFBS regions [14]. The average levels of H3K4me2, H3K27ac, H3K4me3, H3K79me2, and H3K9ac are elevated by 18%, 52%, 19%, 8%, and 31%, respectively, in TFBS areas compared to non-TFBS areas. However, different transcription factors and different cell types have different histone modifications around their respective TFBSs, suggesting that the genomic distribution of histone modifications around TFBSs is transcription factor-specific and cell type-specific [14]. Interestingly, shape and orientation analyses revealed that the distributions of histone modifications around TFBS areas are asymmetric for all chromatin features [12]. This finding suggests that most transcription factor binding events correlate with structured, directional patterns of histone modifications. Moreover, the binding sites of different transcription factors overlap, suggesting the mutual association of transcription factors, and different associations are specific to different genomic contexts. For example, the associations of HDAC2, GABPA, CHD2, GTF2F1, MXI1, and MYC are more specific to promoter regions, while the associations of SP1, EP300, HDAC2, and NANOG are more specific to intergenic regions [12].

Several studies suggest that TFBSs show a polymorphism density that is higher than the average polymorphism density across the human genome [6]. Of all TFBS variants, most overlap one TFBS, while some overlap two or more TFBSs. When we categorized TFBS nucleotide changes by variant type, 95% were single nucleotide polymorphisms, 2% were deletions, and 1% were insertions, suggesting a distribution similar to that of the overall variant types across the human genome (96% are single nucleotide polymorphisms, 2% are deletions, and 1% are insertions) [15]. As regards the genomic locations of TFBS variants, the largest proportion of TFBS variants is found in introns (~50%) and intergenic regions (~30%), with the remaining TFBS variants mostly located in promoters (~10%) and others distributed over coding regions, 5′ untranslated regions (5′UTRs), and 3′ untranslated regions (3′ UTRs) [16]. Similar to the epigenetic signature of TFBSs, TFBS variants are also enriched for various histone modifications (H3K27ac, H3K4me1, and H3K4me3) [17].

## 3. Mechanisms Linking TFBS Variations and Differential Gene Expression

**Figure 1:**
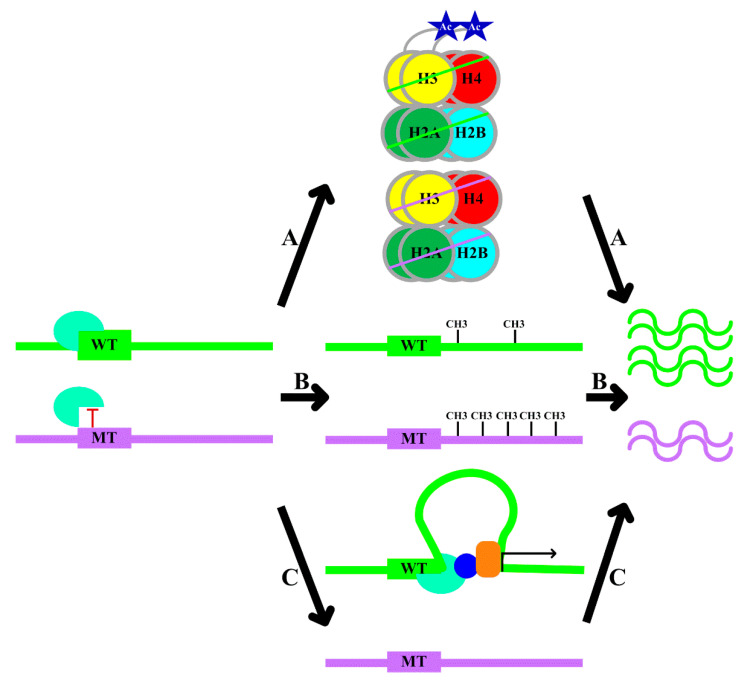
Mechanisms of gene expression regulation by transcription factor binding site (TFBS) variants. Polymorphisms of TFBSs regulate gene expression via several mechanisms: (A) Allele-specific transcription factor binding alters histone modification, which causes differences in gene expression between different alleles. (B) Allele-specific transcription factor binding modulates local DNA methylation, resulting in allele-specific gene expression. (C) Allelic differences introduce differential transcription factor binding, which causes chromatin conformational changes and differential co-factors recruitment (the blue circle and the orange box), leading to differential gene expression. WT: wild-type allele; MT: mutant-type allele.

In light of the functional relevance of TFBS variants in gene regulation, numerous studies have explored the ways in which TFBS variants can regulate gene expression. According to the current literature, three mechanisms through which transcription factors calibrate gene expression have been extensively studied:

A. Local histone modification: transcription factor binding causes specific histone modifications via interactions between transcription factors and chromatin-modifying enzymes [18], and histone modifications regulate gene expression (Figure 1, Pathway A) [19].
B. Local DNA methylation: transcription factor–DNA binding leads to an altered local DNA methylation profile (Figure 1, Pathway B) [20]. Through modulating DNA methylation, transcription factor binding exerts downstream effects on genome regulation. Thus, the consideration of DNA methylation data in the interpretation of the functional role of variants is recommended [20].
C. Changes in chromatin conformation: several studies have utilized chromosome conformation capture (Hi-C) datasets to demonstrate that transcription factors might drive topological genome reorganization and change the structure of enhancer-promoter loops and recruiting other co-factors, thereby contributing to gene regulation (Figure 1, Pathway C) [21].

The allelic variation in TFBSs may affect transcription factor binding [20]. Combined with the aforementioned mechanisms, allele-specific transcription factor binding can result in differential gene expression via the alteration of histone modifications, local DNA methylation, and chromatin conformation. The following findings are in agreement with such arguments:

A. *rs2886870* disrupts a nuclear factor-κB (NF-κB) binding site and is associated with H3K27ac levels and *C3orf59* mRNA expression (Table 1) [22]. *rs4784227* is a breast cancer risk-associated polymorphism. The risk allele of *rs4784227* enhances FOXA1 binding, decreases H3K9Ac levels, inhibits the expression of *TOX3*, and therefore, promotes the proliferation of breast cancer cells (Table 1) [23]. *rs6983267* is associated with numerous malignancies. The risk allele of *rs6983267* is associated with enhanced TCF4 binding and more prominent histone modifications and drives elevated c-*MYC* expression (Table 1) [24].
B. The *rs2240032* allele specifically binds SMAD3, affects the methylation of the promoter region, and influences *RAD50* and *IL4* expression (Table 1) [25]. Similarly, the *rs612529* risk allele decreases binding of YY1 and PU.1, is associated with the hypermethylation of the promoter, specifically downregulates *SIRL-1* expression, and increases the risk of atopic dermatitis (Table 1) [26]. A rare variant at chr22:24,059,610 disrupts the UA4 binding motif, increases the methylation levels at the promoter of the nearby *GUSBP11* gene, and reduces the expression of *GUSBP11* (Table 1) [20].
C. The *rs12913832* risk allele increases the binding of HLTF, LEF1, and MITF to the enhancer region and enhances chromatin loop formation, and increases *OCA2* expression and, thus, pigmentation (Table 1) [27]. The C allele of *rs13228237* causes increased binding of ZNF143, leads to an increase in chromatin loop formation between the first intron of the *ZC3HAV1* gene and two distal regulatory elements, and increases *ZC3HAV1* expression (Table 1) [28]. The presence of the G allele of *rs2802292* creates an HSF1 binding site, which induces promoter–enhancer interaction via chromatin looping, thereby fostering *FOXO3* expression (Table 1) [29].

## 4. Challenges of Investigating Genetic Variants in TFBSs

Although nucleotide changes in TFBSs show great potential as critical players affecting disease characteristics, studies in regard to how such characteristics are actually related to polymorphisms of TFBSs remain rare due to a lack of knowledge in three main areas: (1) deciphering the transcription factors whose binding is affected by genetic variations, (2) elucidating target genes whose expression is modulated by causal polymorphisms, and (3) uncovering the biological consequences of altered target gene expression in diseases.

With regard to the first area, the major challenge is obtaining a complete list of TFBSs from the human genome. Unfortunately, binding site sequence specificity has been analyzed in detail for only a small proportion of transcription factors [30]. ChIP-seq, which has replaced the array-based ChIP-on-chip (ChIP-chip) strategy [31], is the gold-standard method for identifying DNA fragments bound by a specific transcription factor [32]. The newly developed ChIP-exo strategy, in which an exonuclease trims ChIP DNA to a precise distance from the crosslinking site, can result in a single-nucleotide spatial resolution, representing an improvement from the resolution of ChIP-seq [31]. Additionally, there are several complementary methods for studying the interaction between DNA sequences and transcription factors, including electrophoretic mobility shift assay (EMSA), systematic evolution of ligands by exponential enrichment (SELEX) [33], mechanically induced trapping of molecular interactions (MITOMI) [34], and total internal reflection fluorescence (TIRF) [35]. However, not all TFBSs can be easily retrieved with experimental methods because ChIP-grade and/or experiment-grade antibodies are not available for many transcription factors [30]. In addition to experimental methods, there are also several computational approaches for studying the contribution of genetic polymorphisms that create or disrupt TFBSs, such as SNP2TFBS [36], sTRAP [37], MatInspector [38], TFBIND [39], and RSAT [40]. Nevertheless, a single transcription factor can recognize many DNA binding site sequences—from dozens to hundreds—over a range of binding affinities, which are affected by multiple biophysical properties of transcription factors, DNA structure, cooperative cofactors [41], and the methylation statuses of nucleotides [42]. In agreement with this, genetic variants in TFBSs result in changes at the transcript level in specific cell types and induce disease in specific organs [43,44]. Overall, the effects of human TFBS variants depend on the cellular context as well as the local genomic environment. All these factors complicate experimental and in silico analyses of genetic variants that localize within positions in TFBSs [45]. Moreover, there are numerous experimentally characterized TFBSs in the human genome (7–10%) that are derived from repetitive DNA [46], and sequencing reads for repetitive regions tend to be filtered out during analysis [47]. In light of these complexities, it is plausible that our understanding of transcription factor–DNA binding events is informed by only a fraction of the transcription factor–DNA interactions that are biologically active in vivo [48]. In line with this idea, a previous study showed that the results from bioinformatic tools need to be complemented with experimental analyses [37]. There is still ample room for alternative approaches or technologies that will enable a more comprehensive characterization of the current catalog of human TFBSs.

With regard to the second area, a detailed analysis of expression quantitative trait loci (eQTLs) is the most popular approach for identifying associated target genes [49] because a regulatory element could have multiple target genes and genetic variants may not influence the expression of the nearest gene but instead act on distant targets residing kilobases away [50,51]. As transcriptional regulatory networks are highly tissue-specific, eQTL analysis can only be accurately performed within the tissue concerned [52]. However, the target tissue affected by the respective genetic variants might not have been known previously.

Another approach to inferring the effector genes of regulatory regions is the analysis of the spatial chromatin organization by C-methods (chromosome conformation capture-based methods), including ChIA-PET, HiChIP (in situ Hi-C followed by ChIP), and promoter capture Hi-C [50,51]. Although the active regulatory regions are thought to be spatially close to the promoters of their target genes, spatial proximity does not guarantee a functional relationship between a regulatory region and a gene [53,54]. Moreover, while an individual TFBS variant may contribute, neighboring risk variants might also modulate the transcriptional landscape of target genes. Therefore, the relationship suggested from ChIA-PET, HiChIP, or promoter capture Hi-C analyses could arise from neighboring risk variants and not necessarily from the altered transcription factor binding introduced by TFBS variants [55]. Accordingly, C-methods do not replace further functional verification. However, high-throughput versions of C-methods, such as Hi-C, allow for the annotation of all target genes for all potential enhancers from GWAS-identified regions in one experiment. These techniques are often used as intermediate steps for the detection of genes potentially regulated by enhancers in GWAS-identified regions before performing time-consuming functional confirmation [54].

For the third area, assigning biological roles to the identified genes requires experimental validation. In the process of functional validation, overexpression and knockdown experiments are necessary to decipher the biological roles of identified target genes in diseases [56]. The most commonly utilized techniques are RNA activation (RNAa) [57] and RNA interference (RNAi) [58]. CRISPR activation (CRISPRa) and CRISPR interference (CRISPRi) screening are alternative gene-editing approaches [50,51]. However, the following must be noted: (a) It is well documented that, even when we successfully overexpress specific proteins, the expressed proteins do not mimic endogenous proteins in terms of their spatiotemporal expression, localization, and functions [59]. (b) A total of 90–95% of human genes encode two or more isoforms, and different protein isoforms often differ in their structures and biochemical properties and, thus, have distinct functions [59]. In addition, different types of cells may express different splicing isoforms, which add to the diversity in biological function [59]. Although there are some computational models for predicting the functions of isoforms, such as DisoFun [60] and ISOGO [61], experimental evidence is still necessary to functionally validate computational predictions. Functionally validating specific isoforms requires manipulating a specific isoform—without affecting other isoforms—in a cell-specific fashion. This process is very technically challenging [59]. (c) Many genes probably have essential functions; the loss of their function causes lethality, hindering functional validation [62]. (d) Most studies provide information in regard to only the short-term effects of changes in gene expression; longer-term studies may be necessary to provide more comprehensive insights into the effects of altered gene expression on the eventual traits.

## 5. Origin of TFBS Genetic Variants: Natural Selection

As expected from the above understanding, TFBS perturbation leads to strong changes in transcriptional activity throughout development and changes organisms’ phenotypes [63]. One emerging question is why there are so many TFBS genetic variants. Because the natural selection exerted by environmental factors contributes substantially to the population genetic structure [64], one prominent hypothesis is that TFBS variants arise from natural selection and facilitate human adaptation to the local environment. Several theories suggest that TFBS sequences may be particularly important in evolutionary adaptation. The most important reason for this is that variations in such sequences help to minimize the functional tradeoffs associated with evolutionary changes, since these elements primarily impact the expression of a single gene in a specific cell type or under specific conditions, whereas protein-coding variations tend to have broader effects [65]. Compatible with such concepts, whole-genome sequences and genome-wide ChIP and sequencing data demonstrate that natural selection has profoundly influenced human TFBSs since the divergence of humans from chimpanzees 4–6 million years ago. For example, previous analyses estimated that, on average, an adaptive substitution occurred approximately once every ~8300 nucleotides in TFBSs and that approximately one in 20 recent nucleotide substitutions in binding sites have been driven by positive selection, which is much higher than the background substitution rate [65]. Moreover, the local binding affinity of individual binding sites is well correlated with the strength of natural selection at individual binding sites [65]. Additional work has also found that affinity-increasing mutations showed enrichment for adaptive substitutions, whereas affinity-decreasing mutations showed enrichment for weakly deleterious polymorphisms [65]. Furthermore, common low-frequency alleles account for a substantially larger fraction of deleterious mutations in TFBSs than in coding sequences [65]. Positive selection signal acting on TFBSs is also observed [66]. These findings collectively demonstrate the genome-wide impact of natural selection on human TFBSs.

Based on previous observations, it can be concluded that polygenic variations in TFBSs have been a major target of evolutionary forces and a key contributor to different phenotypes across human populations. Numerous well-known examples provide support for this conclusion:

A. Infection: *rs139999735* is associated with *APAF1-interacting protein* (*APIP*), which inhibits pyroptosis and apoptosis, both of which are responses to *Salmonella* infection (Table 2). Individuals homozygous for *rs139999735* show decreased *APIP* expression and, therefore, might generate a better response to *Salmonella* infection. Interestingly, *rs139999735* displays a higher allelic frequency in Africans (0.34) than in Asians (0.11) and Europeans (0.12), suggesting the natural selection of *rs139999735* in Africans [67]. The *ACKR1-*null polymorphism *rs2814778* located in *ACKR1,* which disrupts the binding of the transcription factor GATA binding protein 1 (GATA1), is associated with reduced susceptibility to malaria infections caused by *Plasmodium vivax* (Table 2). The associated protective effects may explain the spread of the *ACKR1-*null polymorphism by natural selection in areas of relatively high malaria transmission, such as central, western, and southeastern Africa, in which the prevalence reaches almost 100% [68]. Another well-studied example is IFN-γ + 874. This risk allele fails to provide a binding site for the transcription factor NF-κB. As NF-κB induces *IFN-γ* expression, the risk allele correlates with reduced *IFN-γ* expression and susceptibility to tuberculosis (Table 2). Because only the more resistant individuals survived and reproduced, over successive generations of selective pressure from tuberculosis, the frequency of the risk genotype decreased, and eventually, the cases of tuberculosis in the white population decreased. Consistent with these observations, the frequency of the risk genotype is much higher in South African populations (47%) than in Sicilian (26%) and Spanish populations (28%) [69].
B. Radiation: *rs201097793* and *rs2279744* both illustrate the molecular adaptation of modern human populations to ultraviolet radiation. *rs201097793* is located in a TFBS and is associated with *MC1R* (Table 2). Interestingly, *rs201097793* has a higher allelic frequency in Africans (0.70) and Asians (0.64) than in Europeans (0.17) [67]. *MC1R* is known to be associated with pigmentation in humans and is maintained by purifying selection in low-latitude, high-ultraviolet-radiation regions, protecting against folate photolysis [70]. In line with this idea, the *rs201097793* allele associated with darker skin pigmentation exhibits a high frequency in Africans and Asians. As regards *rs2279744* (SNP309) in *MDM2*, MDM2 counteracts p53 in a “yin and yang” fashion to regulate embryo implantation [71]. A single-nucleotide change from T to G in *rs2279744* creates a binding site for the transcription factor SP1 [71]. Consistent with this observation, homozygotes for the G allele express more *MDM2* than homozygotes for the T allele [72]. Modern humans migrating northwards to regions with lower ultraviolet radiation required less p53 to avert the adverse effects of p53 hyperactivity, such as embryonic death. Correspondingly, the population data in both East Asia and Europe show that MDM2 *rs2279744* G homozygotes are selected for by low ultraviolet radiation exposure (Table 2) [71].
C. Taste: Taste perception has been critical in evolution, especially for the detection of toxins. *rs139938620* in *TAS1R3*, a sweet receptor, shows a high allelic frequency in Asians (0.79) compared with other populations (Table 2). *TAS1R3* is a component of the dimeric protein *TAS1R1/TAS1R3*, which is the umami taste receptor, and the umami taste is a common feature of many foods in Asia. As a result, it is reasonable to speculate that this variant is beneficial for toxin detection in Asians and is, thus, selected for [67].
D. Water conservation: A well-studied example is *rs16846053*. The minor allele of *rs16846053* in *SLC4A10* that predisposes individuals to increased plasma osmolality—the reduced central sensing of water loss and/or renal water conservation—is underrepresented in the African population (minor allele frequency 0.02) compared with the European population (minor allele frequency 0.10) (Table 2) [73].

## 6. Consequences of TFBS Genetic Variants: Disease Susceptibility

Similar to the selection pressure experienced by TFBS genomic sequences, the genes affecting the risk of gout exhibit the hallmarks of natural selection [74]. Gout is associated with numerous comorbidities, such as increased body mass index [75], chronic kidney disease [76], diabetes, altered low-density lipoprotein (LDL), high-density lipoprotein (HDL), and triglyceride levels [75], coronary artery disease/ischemic heart disease [77,78], hypertension [79], hyperuricemia [75], osteoporosis [80], and prostate cancer [81].

Interestingly, previous studies suggest that there is some selection pressure in these comorbidities (body mass index [82], chronic kidney disease [83], diabetes [84], altered LDL/HDL/triglyceride levels [74,85], coronary artery disease/ischemic heart disease [86], hypertension [87], hyperuricemia [74], osteoporosis [88], and prostate cancer [89]) related to gout [74]. Accordingly, in the following section we focus on examples of mechanistic investigations of the regulatory elements of TFBSs related to transcription factors, affected genes, and disease phenotypes in gout and its associated comorbidities which exhibit selection signatures in susceptibility loci.

### 6.1. Gout

**Figure 2:**
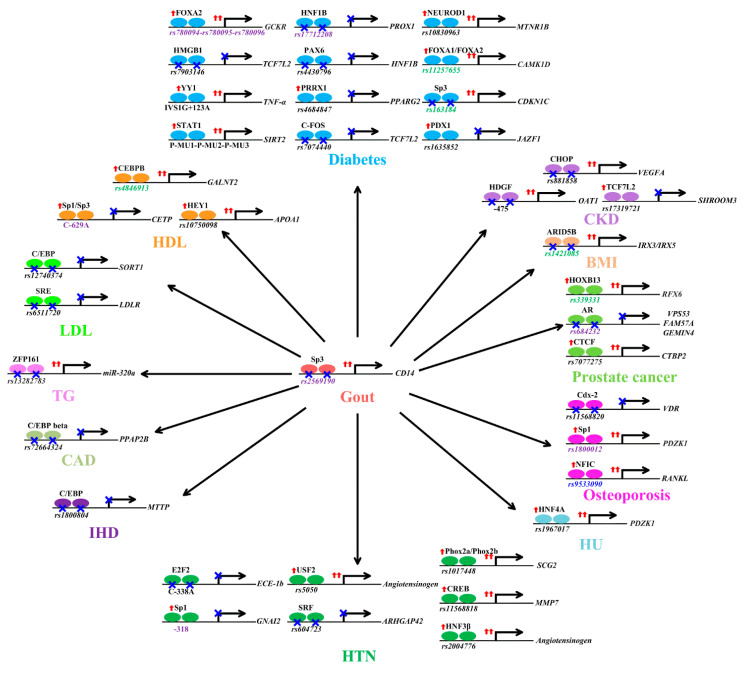
Mechanisms underlying the associations between the risk alleles of various transcription factor binding site (TFBS) variants and susceptibility to gout and related comorbidities (increased body mass index (BMI); chronic kidney disease (CKD); diabetes; levels of high-density lipoprotein (HDL), low-density lipoprotein (LDL), and triglycerides (TGs); coronary artery disease (CAD); ischemic heart disease (IHD); hypertension (HTN); hyperuricemia (HU); osteoporosis; prostate cancer) are shown. Variants (*rs2569190*, *rs17712208*, *rs780094-rs780095-rs780096*, CETP C-629A, GNAI2 -318, *rs1800012*, and *rs684232*) with data supporting a role of histone modification in target gene regulation are shown in purple. Variants (*rs1421085*, *rs11257655*, *rs163184*, *rs4846913*, and *rs339331*) with data suggesting a role of DNA methylation in target gene regulation are shown in green. The variant (*rs9533090*) with data suggesting a role of chromatin conformational changes in target gene regulation is shown in blue. Red arrows indicate increased transcription factor binding or gene expression, while a blue X means decreased transcription factor binding or gene expression.

Gout susceptibility loci displayed selection signatures [74]. In gout, the engagement of CD14 mediates the phagocytosis of monosodium urate crystals by macrophages and their subsequent inflammatory response, culminating in interleukin−1β production [90]. Past studies demonstrate that TFBS DNA variants can directly contribute to gout through altered transcription factor binding. A well-known example is *rs2569190*. The risk allele of *CD14 rs2569190* decreases the affinity of Sp3 protein binding, amplifies the transcriptional activities of *CD14*, and contributes to the development of gout (Figure 2) [91,92,93]. As Sp3 complexes with HDAC to alter histone modification and drive chromatin remodeling [94], it is possible that histone modification is causally related to Sp3 binding-induced *CD14* upregulation.

### 6.2. Body Mass Index

Previous studies found a significant association between gout and increased body mass index, whose genetic loci are under extensive natural selection pressure [75,82]. The current literature also supports the roles of TFBS variants in determining body mass index. A striking example is *rs1421085*. The risk allele of *rs1421085* disrupts a conserved motif in the ARID5B repressor. This disruption results in the derepression of a potent preadipocyte enhancer and the escalation of *IRX3* and *IRX5* expression during adipocyte differentiation [95]. The ultimate consequences of these changes include a cell-autonomous developmental shift from energy-dissipating beige adipocytes to energy-storing white adipocytes [95]. Combined with a reduction in mitochondrial thermogenesis as well as an increase in lipid storage, the risk allele of *rs1421085* ultimately contributes to increased body mass index (Figure 2) [95]. Interestingly, *rs1421085* is located in a linkage disequilibrium block associated with DNA methylation [96], and future study is necessary to reveal the exact role of DNA methylation in the relationship between altered ARID5B binding and *IRX3*/*IRX5* expression.

### 6.3. Chronic Kidney Disease

Gout is associated with chronic kidney disease [76], and selective pressure is also observed for chronic kidney disease-associated alleles [83]. *rs17319721* is associated with chronic kidney disease [97]. The risk allele of *rs17319721* [97] increases the binding of TCF7L2, alters the repressive looping between *rs17319721* and the novel start site, and decreases the expression of a short isoform of *SHROOM3*, which is necessary for kidney function (Figure 2) [98]. *rs881858* is associated with chronic kidney disease [99]. The risk allele of *rs881858* diminishes binding to CHOP [100] and upregulates *VEGFA* expression [44], which is essential for glomerulogenesis and ureteric bud growth during embryogenesis and impacts the number of nephrons (Figure 2) [99]. The risk allele of OAT1-475 decreases the binding of hepatoma-derived growth factor (HDGF) and enhances *OAT1* expression, which results in the increased transportation of organic anion toxins into cells. The cellular accumulation of organic anion toxins causes cytotoxicity and leads to chronic kidney disease (Figure 2) [101]. The roles of histone modification, DNA methylation, and chromatin conformation changes in these chronic kidney disease-associated gene dysregulation remain unexplored.

### 6.4. Diabetes

The association between gout and diabetes is well known [75], and previous studies have uncovered evidence of purifying selection for diabetes-associated variants [84]. Similar to the research related to TFBS variants in gout, several detailed analyses have documented the roles of TFBS genetic variants in diabetes. For example, *rs10830963*, the index variant of the *MTNR1B* locus, overlaps with a NEUROD1 binding site. The risk allele of *rs10830963* specifically binds NEUROD1 and magnifies *MTNR1B* expression, which blocks insulin release from pancreatic *β* cells in response to glucose (Figure 2) [102,103]. Overall, the risk allele of *rs10830963* triggers a cascade of molecular changes facilitating diabetes. The *rs11257655* risk allele shows allele-specific binding to FOXA1/FOXA2, thereby upregulating the transcription of *CAMK1D*, which increases gluconeogenesis and, therefore, the risk of diabetes (Figure 2) [104,105]. The risk allele of *rs11257655* is associated with decreased methylation in the promoter region of *CAMK1D*, supporting the role of DNA methylation in the relationship between the risk allele, FOXA1/FOXA2 binding, and *CAMK1D* expression [84]. *rs163184* is a diabetes susceptibility polymorphism located in the TFBS of Sp3. The risk allele of *rs163184* attenuates Sp3 binding, and therefore, enhances the transcriptional activity of *cyclin-dependent kinase inhibitor 1C* (*CDKN1C*) (Figure 2), which inhibits human *β* cell proliferation and promotes diabetes [106]. Because *rs163184* is associated with nearby CpG differential methylation [107], the likely intermediary role of CpG methylation in Sp3′s regulation of *CDKN1C* expression warrants study. The risk allele of *rs1635852* preferentially recruits PDX1 and lowers *JAZF1* transcription. These alterations could result in impaired β cell function and, therefore, are strongly associated with diabetes (Figure 2) [108]. The risk allele of *rs17712208* disrupts HNF1B binding, decreases H3K27ac, and reduces the expression of *PROX1,* leading to impaired β cell insulin secretion and thereby increasing susceptibility to diabetes (Figure 2) [109,110]. Analogously, the *rs4430796* risk allele decreases PAX6 binding and downregulates the expression of *HNF1B*, leading to an elevated risk of diabetes (Figure 2) [111].

*rs4684847* is another diabetes risk variant located in a TFBS. The *rs4684847* risk allele, by binding the homeobox transcription factor PRRX1, represses *PPARG2*, perturbs lipid metabolism and insulin sensitivity, and contributes to the onset of diabetes (Figure 2) [112]. The *rs7074440* risk allele binds C-FOS with decreased avidity, and thus, attenuates *TCF7L2* expression. The consequence of *TCF7L2* attenuation is aggravated hyperglycemia, which confers susceptibility to diabetes (Figure 2) [113]. FOXA2 also binds an enhancer locus in *GCKR* represented by the haplotype *rs780094*-*rs780095*-*rs780096*. The risk haplotype preferentially binds FOXA2, increases H3K27Ac histone marks, upregulates *GCKR* expression, enhances glucose metabolism, and, therefore, is associated with the risk of diabetes (Figure 2) [114]. *rs7903146* in *TCF7L2* is another common genetic variant highly associated with diabetes [115]. The risk allele of *rs7903146* interferes with HMGB1 binding, leading to reduced *TCF7L2* expression and, therefore, impaired insulin secretion and increased diabetes risk (Figure 2) [115].

IVS1G + 123A is located in a TFBS. The transcription factor YY1 binds allele-specifically to the risk allele of IVS1G + 123A in the *tumor necrosis factor α* (*TNF-α*) gene region and increases *TNF-α* expression and, thereby, diabetes risk (Figure 2) [116]. The risk haplotype of *P*-MU1, *P*-MU2, and *P*-MU3 in the *SIRT2* promoter enhances the binding between signal transducer and activator of transcription 1 (STAT1) and the *SIRT2* promoter, leading to an increase in *SIRT2* transcription, which elevates fasting plasma glucose and glycated hemoglobin (HbA_1c_) (Figure 2) [117]. It remains to be seen whether the transcription factor binding of diabetes-associated variants other than *rs11257655*, *rs163184*, *rs17712208*, and *rs780094*-*rs780095*-*rs780096* regulates target gene expression via altered histone modification, DNA methylation, or chromatin conformational changes (Figure 1).

### 6.5. Dyslipidemia

Gout is associated with an altered lipid profile [75], and risk loci associated with the levels of LDL, HDL, and triglycerides show evident natural selection signatures [74,85]. Several studies have provided evidence for the effects of TFBS variants on lipid metabolism. A well-known example is *rs10750098*. The HDL-increasing allele of *rs10750098* enhances HEY1 binding, elevates the expression of *APOA1*, and increases HDL (Figure 2) [118]. In the same way, *rs4846913*, which is in complete linkage with *rs4846914*, overlaps with CEBPB binding sites (Figure 2) [119]. Functional studies show that the HDL-increasing allele of *rs4846913* strengthens CEBPB binding and is associated with upregulated *GALNT2* [119], which increases HDL levels (Figure 2) [120]. Since *rs4846913/rs4846914* shows associations with DNA methylation [121], the effects of CEBPB on *GALNT2* might be mediated via DNA methylation. Similarly, the HDL-increasing allele of the CETP C-629A polymorphism creates a binding site for the Sp1/Sp3 complex, represses *CETP* promoter transcriptional activity, and therefore increases HDL [122,123]. As a previous study observed that Sp1/Sp3 proteins recruit HDAC complexes to the proximal promoter, thus preventing chromatin remodeling and resulting in the transcriptional repression of the gene, we hypothesize that histone modification may be involved in Sp1/Sp3-induced *CETP* modulation [94].

Similarly, *rs12740374* overlaps with a CCAAT/enhancer binding protein (C/EBP) TFBS. The risk allele of *rs12740374* disrupts C/EBP binding, downregulates the hepatic expression of the *SORT1* gene, and increases the level of LDL (Figure 2) [124,125]. Another example is *rs6511720.* The risk allele of * rs6511720* inhibits serum response element (SRE) binding, attenuates *LDLR* expression, and increases LDL (Figure 2) [126]. One more example is *rs13282783*, which is associated with hypertriglyceridemia [127]. Notably, the region around *rs13282783* overlaps with the ZFP161 binding site [127]. Because the *rs13282783* risk allele prevents ZFP161 binding, the risk allele upregulates miR-320a, which aggravates hypertriglyceridemia (Figure 2) [127].

### 6.6. Heart Disease

Gout is associated with increased coronary artery disease [77] and ischemic heart disease [78], which show selection signatures on trait-associated variants [86]. A series of studies provide solid evidence that DNA variants in TFBSs, including *rs72664324* and *rs1800804*, influence coronary artery disease/ischemic heart disease via allele-specific transcription factor binding.

The coronary artery disease risk allele of *rs72664324* decreases C/EBP beta binding and inhibits the expression of *PPAP2B,* a gene that deactivates proinflammatory mediators, thus promoting coronary artery disease (Figure 2) [128]. The *rs1800804* risk allele has a weak affinity for C/EBP binding, lowers *MTTP* transcriptional activity, and results in a higher accumulation of lipids and increased susceptibility to ischemic heart disease (Figure 2) [129]. Whether histone modification, DNA methylation, and chromatin conformation play mediating roles in the relationship between transcription factor binding and these target genes’ expression remains unexplored.

### 6.7. Hypertension

Hypertension is present in a considerable number of patients with gout [79], and selection pressures on hypertension-associated loci have been reported in the past [87]. Several studies further provide evidence for the effects of TFBS variants on the risk of hypertension. The risk allele of *rs1017448* binds to Phox2a and Phox2b in an allele-dependent manner to enhance *SCG2* expression [130,131]. The secretoneurin peptide derived from *SCG2* stimulates the migration and proliferation of vascular smooth muscle cells and acts as an endothelial cytokine promoting angiogenesis and vasculogenesis, facilitating an increase in blood pressure [131]. Therefore, the risk allele of *rs1017448* confers hypertension susceptibility (Figure 2) [131]. Through transfection experiments, computational prediction, and structure-based conformational and molecular dynamics simulation studies, it has been shown that the risk allele of *rs11568818* exhibits increased binding affinity for cyclic AMP response element-binding protein (CREB), confers increased promoter activity, and enhances *matrix metalloproteinase-7* (*MMP7*) expression, which aggravates hypertension (Figure 2) [132]. Another example is *rs2004776*, which is located in HNF3β binding sites. The risk allele of *rs2004776* binds HNF3β more strongly than does the non-risk allele, increasing angiotensinogen expression and thereby aggravating hypertension (Figure 2) [133]. Similarly, *rs5050* in the angiotensinogen promoter overlaps with the binding sites of upstream stimulatory factor 2 (USF2) [134]. The risk allele of *rs5050* preferentially binds USF2, augments angiotensinogen transcription, and is associated with hypertension (Figure 2) [134,135]. Additionally, the DNA sequence around *rs604723*, a hypertension-associated polymorphism, matches the serum response factor (SRF) binding site [136]. The risk allele variation at *rs604723* decreases SRF binding, downregulates *ARHGAP42* expression, and increases Ras homologue family member A (RhoA)-dependent vascular smooth muscle cell contractility, thereby contributing to hypertension risk (Figure 2) [136,137]. Likewise, the ECE1 C-338A polymorphism is strongly associated with hypertension [138]. The risk allele of the ECE1 C-338A polymorphism interferes with E2F2 binding and endothelial ECE-1b expression and facilitates hypertension (Figure 2) [138].

The risk allele of the GNAI2-318 specifically binds Sp1 and reduces *GNAI2* expression [139]. *GNAI2* downregulation promotes sodium retention, sympathoexcitation, and rapid renal nerve-dependent hypertension [140]. The risk allele of GNAI2-318 impairs transcriptional activity through the specific binding of Sp1, exacerbating salt-sensitive hypertension via a renal nerve-dependent mechanism (Figure 2) [140]. As the Sp1 protein interacts with HDAC complexes to modify histone acetylation, we hypothesize that histone modification may be involved in Sp1-mediated *GNAI2* downregulation [94]. Except for GNAI2-318, there are no clues about the roles of histone modification, DNA methylation, and chromatin conformation changes in target gene regulation by these hypertension-associated TFBS variants.

### 6.8. Hyperuricemia

Gout is associated with hyperuricemia [141], and uric acid-associated variants harbor evidence of selection [74]. Similar to the findings in regard to TFBS variants in gout, studies also support a role of TFBS nucleotide variation in hyperuricemia. The urate-increasing allele of *rs1967017* enhances HNF4A’s binding to the *PDZK1* promoter, thereby stimulating *PDZK1* expression (Figure 2) [142]. As PDZK1 is a scaffold protein for many ion-channel transporters and PDZK1 increases the apical localization of ABCG2, a urate transporter in the intestine [143], increased PDZK1 expression can increase urate absorption and thereby contribute to hyperuricemia. Unfortunately, the molecular mechanism that results in HNF4A-induced PDZK1 expression changes is not known.

### 6.9. Osteoporosis

Gout is associated with osteoporosis [80], which is under the forces of purifying selection [88]. *rs11568820* is associated with osteoporosis [144]. The risk allele markedly decreases the binding of Cdx-2 compared with that for non-risk alleles and suppresses the transcriptional activity of the *VDR*, which plays a key role in intestinal calcium absorption and the development of osteoporosis [145,146]. *rs1800012* is significantly associated with osteoporosis. The *COL1A1* *rs1800012* risk allele increases the binding affinity of Sp1 protein, increases the ratio of *COL1A1* to *COL1A2*, and reduces the yield strength of bone [147,148]. As the Sp1 protein results in histone modification [94], histone modification is one potential mediator of Sp1-mediated *COL1A1* dysregulation. *rs9533090* is an allele-specific regulatory polymorphism associated with osteoporosis [149,150]. The risk allele robustly recruits transcription factor NFIC and increases *RANKL* expression, thus, contributing to osteoporosis risk [149]. Because *rs9533090* forms a long-range chromatin interaction with *RANKL* to regulate *RANKL* expression, chromatin conformational changes possibly mediate the link between risk allele and osteoporosis susceptibility [149].

### 6.10. Prostate Cancer

Gout is associated with prostate cancer [81], and prostate cancer risk genes display the signature of selection pressure [89]. Several studies have also identified the transcription factor and downstream effector genes involved with the prostate cancer-associated TFBS variant. The prostate cancer-associated polymorphism *rs339331* lies within a functional HOXB13-binding site. The risk-associated allele at *rs339331* increases HOXB13 binding in a transcriptional enhancer, resulting in the allele-specific upregulation of *RFX6*, which enhances prostate cancer cell proliferation, migration, and invasion [151]. Because *rs339331* is associated with CpG methylation [152], DNA methylation might mediate the regulatory chain between HOXB13 and *RFX6*. The prostate cancer risk polymorphism *rs684232* has been reported to function as an eQTL. The risk allele diminishes androgen receptor (AR) occupancy, is associated with decreased H3K27ac levels, and downregulates the expression of *VPS53*, *FAM57A*, and *GEMIN4*, and the knockdown of *VPS53*, *FAM57A*, and *GEMIN4* in prostate cancer cells results in an increase in cell viability [153]. For prostate cancer-associated *rs7077275* [154], the risk allele enhances CTCF binding and enhances the allele-specific expression of *CTBP2* [155], which decreases the apoptosis of prostate cancer cells and increases tumor growth in a mouse xenograft model of human prostate cancer [156]. It is unknown about the role of histone modification, DNA methylation, and chromatin conformational changes in the link between CTCF and *CTBP2* expression.

## 7. Consequences of TFBS Genetic Variants: Treatment Response

In addition to affecting disease susceptibility, TFBSs also induce functional effects, including modulating the responses to drugs [157]. A growing body of work also implicates variants of TFBSs in the treatment responses of patients with increased body mass index and coronary artery disease. The first example is CYP2C19*17 (*rs12248560*). Clopidogrel is often used as part of dual antiplatelet therapy for the secondary prevention of acute coronary syndrome [158]. The CYP2C19*17 allele creates a consensus binding site for the GATA transcription factor family, resulting in increased *CYP2C19* expression and activity [159]. Interestingly, the CYP2C19*17 allele is associated with a better platelet response to clopidogrel in acute coronary syndrome patients [160]. Another example is GNAS-1211. The G allele at position-1211 of the *GNAS* promoter results in enhanced upstream stimulatory factor 1 binding and upregulated Gαs expression and lipolysis. In line with these findings, the effect on body weight change in response to sibutramine was stronger in G-allele carriers than in carriers of other alleles in a clinical phase 3 trial [161]. The intermediatory roles of histone modification, DNA methylation, and chromatin conformational changes in the target gene regulation induced by these two polymorphisms remain unexplored.

## 8. Conclusions

Although genetic variants of TFBSs have offered humans survival advantages in their fight against nature during evolution, they have also resulted in predispositions to diseases in modern environments. In other words, TFBS variants are not only triggered by human adaptation to evolution but are also triggers of various diseases. Based on this viewpoint, TFBS variants could be considered a genomic defense mechanism against potential environmental threats, contributing to genetic diversity and effective adaptation. However, various diseases are the price humans pay for such adaptation and survival.

Despite the well-known fact that the genetic variants located in TFBSs constitute important mediators of phenotypes, there are relatively few examples that establish a clear mechanistic relationship between causal variants, involved transcription factors, and implicated downstream genes. Furthermore, the current understanding of the effects of transcription factor binding-induced histone modification, DNA methylation, and chromatin conformational changes is extremely limited. A major obstacle is the inherently complex relationship between the genomic sequence, the associated histone and methylation profile and chromatin structure, and the involved transcription factors and target genes. Deciphering the regulatory “logic” underlying TFBS variation, the effects on target genes, the ultimate biological consequences, and the role of these three mechanisms in regulatory pathways remains one of the greatest challenges facing the genomics field today. New experimental and computational approaches that enable us to better predict genuinely involved transcription factors and affected downstream genes and evaluate how motif variation affects transcription factor–DNA binding are urgently needed. Although there is still a long road ahead, progress has been made in dissecting the genetic basis of transcription factor–DNA binding variation. It is our hope that these efforts will endow us with a nucleotide-level understanding of various molecular mechanisms underlying numerous complex traits and the ability to ultimately translate these findings into new therapies.

## Figures and Tables

**Table 1:** Examples of TFBS variants that alter transcription factor binding, regulating target gene expression through altered histone modification, DNA methylation, and chromatin conformational changes.

Variants	Transcription Factors	Target Genes	References
Alter histone modification			
*rs2886870*	NF-κB	*C3orf59*	[22]
*rs4784227*	FOXA1	* TOX3 *	[23]
* rs6983267 *	TCF4	c-*MYC*	[24]
Alter DNA methylation			
*rs2240032*	SMAD3	*RAD50* and *IL4*	[25]
*rs612529*	YY1 and PU.1	*SIRL-1*	[26]
chr22:24,059,610	UA4	*GUSBP11*	[20]
Alter chromatin conformation			
*rs12913832*	HLTF, LEF1, and MITF	*OCA2*	[27]
*rs13228237*	ZNF143	*ZC3HAV1*	[28]
*rs2802292*	HSF1	*FOXO3*	[29]

**Table 2:** Examples of selective pressures acting on TFBSs.

Category	Variant	Gene	Biological Function	Reference
Infection	* rs139999735 *	* APIP *	Response to *Salmonella*	[67]
	* rs281477 * 8	* ACKR1 *	Protection against malaria infection	[68]
	IFN-γ + 874	IFN-γ	Tuberculosis susceptibility	[69]
Radiation	* rs201097793 *	* MC1R *	Pigmentation	[67]
	* rs2279744 *	* MDM2 *	Embryo implantation	[71,72]
Taste	* rs139938620 *	* TAS1R3 *	Umami taste	[67]
Water conservation	*rs16846053*	*SLC4A10*	Increased plasma osmolality	[73]

## Data Availability

No new data were created or analyzed in this study. Data sharing is not applicable to this article.

## References

[1] Hindorff L.A., Sethupathy P., Junkins H.A., Ramos E.M., Mehta J.P., Collins F.S., Manolio T.A. (2009). Potential etiologic and functional implications of genome-wide association loci for human diseases and traits. Proc. Natl. Acad. Sci. USA.

[2] Ponomarenko M., Rasskazov D., Chadaeva I., Sharypova E., Drachkova I., Oshchepkov D., Ponomarenko P., Savinkova L., Oshchepkova E., Nazarenko M. (2020). Candidate SNP Markers of Atherogenesis Significantly Shifting the Affinity of TATA-Binding Protein for Human Gene Promoters show stabilizing Natural Selection as a Sum of Neutral Drift Accelerating Atherogenesis and Directional Natural Selection Slowing It. Int. J. Mol. Sci..

[3] Wang X., Zhou T., Wunderlich Z., Maurano M.T., DePace A.H., Nuzhdin S.V., Rohs R. (2018). Analysis of Genetic Variation Indicates DNA Shape Involvement in Purifying Selection. Mol. Biol. Evol..

[4] Suter D.M. (2020). Transcription Factors and DNA Play Hide and Seek. Trends. Cell. Biol..

[5] Johnston A.D., Simões-Pires C.A., Thompson T.V., Suzuki M., Greally J.M. (2019). Functional genetic variants can mediate their regulatory effects through alteration of transcription factor binding. Nat. Commun..

[6] Graae L., Paddock S., Belin A.C. (2015). ReMo-SNPs: A new software tool for identification of polymorphisms in regions and motifs genome-wide. Genet. Res. (Camb.).

[7] Tuğrul M., Paixão T., Barton N.H., Tkačik G. (2015). Dynamics of Transcription Factor Binding Site Evolution. Plos. Genet..

[8] Nishizaki S.S., Ng N., Dong S., Porter R.S., Morterud C., Williams C., Asman C., Switzenberg J.A., Boyle A.P. (2020). Predicting the effects of SNPs on transcription factor binding affinity. Bioinformatics.

[9] Auton A., Brooks L.D., Durbin R.M., Garrison E.P., Kang H.M., Korbel J.O., Marchini J.L., McCarthy S., McVean G.A., Abecasis G.R. (2015). A global reference for human genetic variation. Nature.

[10] Yan J., Qiu Y., Ribeiro Dos Santos A.M., Yin Y., Li Y.E., Vinckier N., Nariai N., Benaglio P., Raman A., Li X. (2021). Systematic analysis of binding of transcription factors to noncoding variants. Nature.

[11] Kasowski M., Grubert F., Heffelfinger C., Hariharan M., Asabere A., Waszak S.M., Habegger L., Rozowsky J., Shi M., Urban A.E. (2010). Variation in transcription factor binding among humans. Science.

[12] Consortium E.P. (2012). An integrated encyclopedia of DNA elements in the human genome. Nature.

[13] Samee M.A.H., Bruneau B.G., Pollard K.S. (2019). A De Novo Shape Motif Discovery Algorithm Reveals Preferences of Transcription Factors for DNA Shape Beyond Sequence Motifs. Cell. Syst..

[14] Xin B., Rohs R. (2018). Relationship between histone modifications and transcription factor binding is protein family specific. Genome. Res..

[15] Eickhardt E., Damm A.T., Grove J., Boerglum A.D., Lescai F. (2016). Estimating the functional impact of INDELs in transcription factor binding sites: A genome-wide landscape. bioRxiv..

[16] de Santiago I., Liu W., Yuan K., O’Reilly M., Chilamakuri C.S., Ponder B.A., Meyer K.B., Markowetz F. (2017). BaalChIP: Bayesian analysis of allele-specific transcription factor binding in cancer genomes. Genome. Biol..

[17] Kasowski M., Kyriazopoulou-Panagiotopoulou S., Grubert F., Zaugg J.B., Kundaje A., Liu Y., Boyle A.P., Zhang Q.C., Zakharia F., Spacek D.V. (2013). Extensive variation in chromatin states across humans. Science.

[18] Benveniste D., Sonntag H.J., Sanguinetti G., Sproul D. (2014). Transcription factor binding predicts histone modifications in human cell lines. Proc. Natl. Acad. Sci. USA.

[19] Lawrence M., Daujat S., Schneider R. (2016). Lateral Thinking: How Histone Modifications Regulate Gene Expression. Trends. Genet..

[20] Martin-Trujillo A., Patel N., Richter F., Jadhav B., Garg P., Morton S.U., McKean D.M., DePalma S.R., Goldmuntz E., Gruber D. (2020). Rare genetic variation at transcription factor binding sites modulates local DNA methylation profiles. PLoS Genet..

[21] Di Giammartino D.C., Polyzos A., Apostolou E. (2020). Transcription factors: Building hubs in the 3D space. Cell. Cycle.

[22] McVicker G., van de Geijn B., Degner J.F., Cain C.E., Banovich N.E., Raj A., Lewellen N., Myrthil M., Gilad Y., Pritchard J.K. (2013). Identification of genetic variants that affect histone modifications in human cells. Science.

[23] Cowper-Sal lari R., Zhang X., Wright J.B., Bailey S.D., Cole M.D., Eeckhoute J., Moore J.H., Lupien M. (2012). Breast cancer risk-associated SNPs modulate the affinity of chromatin for FOXA1 and alter gene expression. Nat. Genet..

[24] Wright J.B., Brown S.J., Cole M.D. (2010). Upregulation of c-MYC in cis through a large chromatin loop linked to a cancer risk-associated single-nucleotide polymorphism in colorectal cancer cells. Mol. Cell. Biol..

[25] Schieck M., Sharma V., Michel S., Toncheva A.A., Worth L., Potaczek D.P., Genuneit J., Kretschmer A., Depner M., Dalphin J.C. (2014). A polymorphism in the TH 2 locus control region is associated with changes in DNA methylation and gene expression. Allergy.

[26] Kumar D., Puan K.J., Andiappan A.K., Lee B., Westerlaken G.H., Haase D., Melchiotti R., Li Z., Yusof N., Lum J. (2017). A functional SNP associated with atopic dermatitis controls cell type-specific methylation of the VSTM1 gene locus. Genome Med..

[27] Visser M., Kayser M., Palstra R.J. (2012). HERC2 rs12913832 modulates human pigmentation by attenuating chromatin-loop formation between a long-range enhancer and the OCA2 promoter. Genome Res..

[28] Bailey S.D., Zhang X., Desai K., Aid M., Corradin O., Cowper-Sal Lari R., Akhtar-Zaidi B., Scacheri P.C., Haibe-Kains B., Lupien M. (2015). ZNF143 provides sequence specificity to secure chromatin interactions at gene promoters. Nat. Commun..

[29] Sanese P., Forte G., Disciglio V., Grossi V., Simone C. (2019). FOXO3 on the Road to Longevity: Lessons from SNPs and Chromatin Hubs. Comput. Struct. Biotechnol. J..

[30] Lambert S.A., Jolma A., Campitelli L.F., Das P.K., Yin Y., Albu M., Chen X., Taipale J., Hughes T.R., Weirauch M.T. (2018). The Human Transcription Factors. Cell.

[31] Ma T., Ye Z., Wang L. (2019). Genome Wide Approaches to Identify Protein-DNA Interactions. Curr. Med. Chem..

[32] Xu J., Kudron M.M., Victorsen A., Gao J., Ammouri H.N., Navarro F.C.P., Gevirtzman L., Waterston R.H., White K.P., Reinke V. (2021). To mock or not: A comprehensive comparison of mock IP and DNA input for ChIP-seq. Nucleic. Acids. Res..

[33] Yadav M., Singh R.S., Hogan D., Vidhyasagar V., Yang S., Yeuk Wah Chung I., Kusalik A., Dmitriev O.Y., Cygler M., Wu Y. (2020). The KH domain facilitates the substrate specificity and unwinding processivity of DDX43 helicase. J. Biol. Chem..

[34] Shokri L., Inukai S., Hafner A., Weinand K., Hens K., Vedenko A., Gisselbrecht S.S., Dainese R., Bischof J., Furger E. (2019). A Comprehensive Drosophila melanogaster Transcription Factor Interactome. Cell. Rep..

[35] Horn A.E., Kugel J.F., Goodrich J.A. (2016). Single molecule microscopy reveals mechanistic insight into RNA polymerase II preinitiation complex assembly and transcriptional activity. Nucleic. Acids. Res..

[36] Kubota N., Suyama M. (2020). An integrated analysis of public genomic data unveils a possible functional mechanism of psoriasis risk via a long-range ERRFI1 enhancer. BMC. Med. Genom..

[37] Moradifard S., Saghiri R., Ehsani P., Mirkhani F., Ebrahimi-Rad M. (2020). A preliminary computational outputs versus experimental results: Application of sTRAP, a biophysical tool for the analysis of SNPs of transcription factor-binding sites. Mol. Genet. Genomic. Med..

[38] Stalke A., Pfister E.D., Baumann U., Illig T., Reischl E., Sandbothe M., Vajen B., Huge N., Schlegelberger B., von Neuhoff N. (2020). MTF1 binds to metal-responsive element e within the ATP7B promoter and is a strong candidate in regulating the ATP7B expression. Ann. Hum. Genet..

[39] de Smith A.J., Walsh K.M., Francis S.S., Zhang C., Hansen H.M., Smirnov I., Morimoto L., Whitehead T.P., Kang A., Shao X. (2018). BMI1 enhancer polymorphism underlies chromosome 10p12.31 association with childhood acute lymphoblastic leukemia. Int. J. Cancer..

[40] Santana-Garcia W., Rocha-Acevedo M., Ramirez-Navarro L., Mbouamboua Y., Thieffry D., Thomas-Chollier M., Contreras-Moreira B., van Helden J., Medina-Rivera A. (2019). RSAT variation-tools: An accessible and flexible framework to predict the impact of regulatory variants on transcription factor binding. Comput. Struct. Biotechnol. J..

[41] Siggers T., Gordân R. (2014). Protein-DNA binding: Complexities and multi-protein codes. Nucleic. Acids. Res..

[42] Zuo Z., Roy B., Chang Y.K., Granas D., Stormo G.D. (2017). Measuring quantitative effects of methylation on transcription factor-DNA binding affinity. Sci. Adv..

[43] Pai A.A., Pritchard J.K., Gilad Y. (2015). The genetic and mechanistic basis for variation in gene regulation. PLoS Genet..

[44] Ledo N., Ko Y.A., Park A.S., Kang H.M., Han S.Y., Choi P., Susztak K. (2015). Functional genomic annotation of genetic risk loci highlights inflammation and epithelial biology networks in CKD. J. Am. Soc. Nephrol..

[45] Maurano M.T., Haugen E., Sandstrom R., Vierstra J., Shafer A., Kaul R., Stamatoyannopoulos J.A. (2015). Large-scale identification of sequence variants influencing human transcription factor occupancy in vivo. Nat. Genet..

[46] Polavarapu N., Mariño-Ramírez L., Landsman D., McDonald J.F., Jordan I.K. (2008). Evolutionary rates and patterns for human transcription factor binding sites derived from repetitive DNA. BMC Genom..

[47] Amemiya H.M., Kundaje A., Boyle A.P. (2019). The ENCODE Blacklist: Identification of Problematic Regions of the Genome. Sci. Rep..

[48] Cusanovich D.A., Pavlovic B., Pritchard J.K., Gilad Y. (2014). The functional consequences of variation in transcription factor binding. PLoS. Genet..

[49] Liu Y., Liu X., Zheng Z., Ma T., Long H., Cheng H., Fang M., Gong J., Li X., Zhao S. (2020). Genome-wide analysis of expression QTL (eQTL) and allele-specific expression (ASE) in pig muscle identifies candidate genes for meat quality traits. Genet. Sel. Evol..

[50] Mumbach M.R., Satpathy A.T., Boyle E.A., Dai C., Gowen B.G., Cho S.W., Nguyen M.L., Rubin A.J., Granja J.M., Kazane K.R. (2017). Enhancer connectome in primary human cells identifies target genes of disease-associated DNA elements. Nat. Genet..

[51] Miguel-Escalada I., Bonàs-Guarch S., Cebola I., Ponsa-Cobas J., Mendieta-Esteban J., Atla G., Javierre B.M., Rolando D.M.Y., Farabella I., Morgan C.C. (2019). Human pancreatic islet three-dimensional chromatin architecture provides insights into the genetics of type 2 diabetes. Nat. Genet..

[52] Ballester B., Medina-Rivera A., Schmidt D., Gonzàlez-Porta M., Carlucci M., Chen X., Chessman K., Faure A.J., Funnell A.P., Goncalves A. (2014). Multi-species, multi-transcription factor binding highlights conserved control of tissue-specific biological pathways. Elife.

[53] Splinter E., Heath H., Kooren J., Palstra R.J., Klous P., Grosveld F., Galjart N., de Laat W. (2006). CTCF mediates long-range chromatin looping and local histone modification in the beta-globin locus. Genes Dev..

[54] Golov A.K., Kondratyev N.V., Kostyuk G.P., Golimbet A.V.E. (2020). Novel Approaches for Identifying the Molecular Background of Schizophrenia. Cells.

[55] Hu M., Cherkaoui I., Misra S., Rutter G.A. (2020). Functional Genomics in Pancreatic β Cells: Recent Advances in Gene Deletion and Genome Editing Technologies for Diabetes Research. Front. Endocrinol. (Lausanne.).

[56] Lillian J., Tessema L., Gessner R., Wilson C., Kandl K. (2020). Utilizing Knockdowns, Overexpression and Tagging to Study the SUMO Proteases Ulp1 and Ulp2 in *Tetrahymena thermophila*. FASEB J..

[57] Kwok A., Raulf N., Habib N. (2019). Developing small activating RNA as a therapeutic: Current challenges and promises. Ther. Deliv..

[58] Lu Z.R., Laney V.E.A., Hall R., Ayat N. (2021). Environment-Responsive Lipid/siRNA Nanoparticles for Cancer Therapy. Adv. Healthc. Mater..

[59] Liu H., Pizzano S., Li R., Zhao W., Veling M.W., Hu Y., Yang L., Ye B. (2020). isoTarget: A Genetic Method for Analyzing the Functional Diversity of Splicing Isoforms in Vivo. Cell. Rep..

[60] Wang K., Wang J., Domeniconi C., Zhang X., Yu G. (2020). Differentiating isoform functions with collaborative matrix factorization. Bioinformatics.

[61] Ferrer-Bonsoms J.A., Cassol I., Fernández-Acín P., Castilla C., Carazo F., Rubio A. (2020). ISOGO: Functional annotation of protein-coding splice variants. Sci. Rep..

[62] Derks M.F.L., Gjuvsland A.B., Bosse M., Lopes M.S., van Son M., Harlizius B., Tan B.F., Hamland H., Grindflek E., Groenen M.A.M. (2019). Loss of function mutations in essential genes cause embryonic lethality in pigs. PLoS Genet..

[63] Le D.D., Shimko T.C., Aditham A.K., Keys A.M., Longwell S.A., Orenstein Y., Fordyce P.M. (2018). Comprehensive, high-resolution binding energy landscapes reveal context dependencies of transcription factor binding. Proc. Natl. Acad. Sci. USA.

[64] Li L.F., Cushman S.A., He Y.X., Li Y. (2020). Genome sequencing and population genomics modeling provide insights into the local adaptation of weeping forsythia. Hortic. Res..

[65] Arbiza L., Gronau I., Aksoy B.A., Hubisz M.J., Gulko B., Keinan A., Siepel A. (2013). Genome-wide inference of natural selection on human transcription factor binding sites. Nat. Genet..

[66] Moyerbrailean G.A., Kalita C.A., Harvey C.T., Wen X., Luca F., Pique-Regi R. (2016). Which Genetics Variants in DNase-Seq Footprints Are More Likely to Alter Binding?. PLoS Genet..

[67] Ribeiro-dos-Santos A.M., da Silva V.L., de Souza J.E., de Souza S.J. (2015). Populational landscape of INDELs affecting transcription factor-binding sites in humans. BMC Genom..

[68] Rappoport N., Simon A.J., Amariglio N., Rechavi G. (2019). The Duffy antigen receptor for chemokines, ACKR1,- ‘Jeanne DARC’ of benign neutropenia. Br. J. Haematol..

[69] Tso H.W., Ip W.K., Chong W.P., Tam C.M., Chiang A.K., Lau Y.L. (2005). Association of interferon gamma and interleukin 10 genes with tuberculosis in Hong Kong Chinese. Genes Immun..

[70] Jablonski N.G., Chaplin G. (2010). Colloquium paper: Human skin pigmentation as an adaptation to UV radiation. Proc. Natl. Acad. Sci. USA.

[71] Shi H., Su B. (2010). Molecular adaptation of modern human populations. Int. J. Evol. Biol..

[72] Bond G.L., Hu W., Bond E.E., Robins H., Lutzker S.G., Arva N.C., Bargonetti J., Bartel F., Taubert H., Wuerl P. (2004). A single nucleotide polymorphism in the MDM2 promoter attenuates the p53 tumor suppressor pathway and accelerates tumor formation in humans. Cell.

[73] Böger C.A., Gorski M., McMahon G.M., Xu H., Chang Y.C., van der Most P.J., Navis G., Nolte I.M., de Borst M.H., Zhang W. (2017). NFAT5 and SLC4A10 Loci Associate with Plasma Osmolality. J. Am. Soc. Nephrol..

[74] Okada Y., Momozawa Y., Sakaue S., Kanai M., Ishigaki K., Akiyama M., Kishikawa T., Arai Y., Sasaki T., Kosaki K. (2018). Deep whole-genome sequencing reveals recent selection signatures linked to evolution and disease risk of Japanese. Nat. Commun..

[75] Chen C.J., Tseng C.C., Yen J.H., Chang J.G., Chou W.C., Chu H.W., Chang S.J., Liao W.T. (2018). ABCG2 contributes to the development of gout and hyperuricemia in a genome-wide association study. Sci. Rep..

[76] Roughley M., Sultan A.A., Clarson L., Muller S., Whittle R., Belcher J., Mallen C.D., Roddy E. (2018). Risk of chronic kidney disease in patients with gout and the impact of urate lowering therapy: A population-based cohort study. Arthritis. Res. Ther..

[77] Huang W.S., Lin C.L., Tsai C.H., Chang K.H. (2020). Association of gout with CAD and effect of antigout therapy on CVD risk among gout patients. J. Investig. Med..

[78] Thanassoulis G., Brophy J.M., Richard H., Pilote L. (2010). Gout, allopurinol use, and heart failure outcomes. Arch. Intern. Med..

[79] Borghi C., Virdis A. (2019). Serum Urate, Uricase, and Blood Pressure Control in Gout. Hypertension.

[80] Kok V.C., Horng J.T., Wang M.N., Chen Z.Y., Kuo J.T., Hung G.D. (2018). Gout as a risk factor for osteoporosis: Epidemiologic evidence from a population-based longitudinal study involving 108,060 individuals. Osteoporos. Int..

[81] Shih H.J., Kao M.C., Tsai P.S., Fan Y.C., Huang C.J. (2017). Long-term allopurinol use decreases the risk of prostate cancer in patients with gout: A population-based study. Prostate. Cancer. Prostatic. Dis..

[82] Sanjak J.S., Sidorenko J., Robinson M.R., Thornton K.R., Visscher P.M. (2018). Evidence of directional and stabilizing selection in contemporary humans. Proc. Natl. Acad. Sci. USA.

[83] Devuyst O., Pattaro C. (2018). The UMOD Locus: Insights into the Pathogenesis and Prognosis of Kidney Disease. J. Am. Soc. Nephrol..

[84] Xue A., Wu Y., Zhu Z., Zhang F., Kemper K.E., Zheng Z., Yengo L., Lloyd-Jones L.R., Sidorenko J., McRae A.F. (2018). Genome-wide association analyses identify 143 risk variants and putative regulatory mechanisms for type 2 diabetes. Nat. Commun..

[85] Dobon B., Montanucci L., Peretó J., Bertranpetit J., Laayouni H. (2019). Gene connectivity and enzyme evolution in the human metabolic network. Biol. Direct..

[86] Byars S.G., Huang Q.Q., Gray L.A., Bakshi A., Ripatti S., Abraham G., Stearns S.C., Inouye M. (2017). Genetic loci associated with coronary artery disease harbor evidence of selection and antagonistic pleiotropy. PLoS Genet..

[87] Hoh B.P., Abdul Rahman T., Yusoff K. (2019). Natural selection and local adaptation of blood pressure regulation and their perspectives on precision medicine in hypertension. Hereditas.

[88] Mullin B.H., Walsh J.P., Zheng H.F., Brown S.J., Surdulescu G.L., Curtis C., Breen G., Dudbridge F., Richards J.B., Spector T.D. (2016). Genome-wide association study using family-based cohorts identifies the WLS and CCDC170/ESR1 loci as associated with bone mineral density. BMC Genom..

[89] Bányai L., Trexler M., Kerekes K., Csuka O., Patthy L. (2021). Use of signals of positive and negative selection to distinguish cancer genes and passenger genes. Elife.

[90] Wu M., Tian Y., Wang Q., Guo C. (2020). Gout: A disease involved with complicated immunoinflammatory responses: A narrative review. Clin. Rheumatol..

[91] McKinney C., Stamp L.K., Dalbeth N., Topless R.K., Day R.O., Kannangara D.R., Williams K.M., Janssen M., Jansen T.L., Joosten L.A. (2015). Multiplicative interaction of functional inflammasome genetic variants in determining the risk of gout. Arthritis. Res. Ther..

[92] Zhu W., Deng Y., Zhou X. (2018). Multiple Membrane Transporters and Some Immune Regulatory Genes are Major Genetic Factors to Gout. Open. Rheumatol. J..

[93] Munthe-Kaas M.C., Torjussen T.M., Gervin K., Lødrup Carlsen K.C., Carlsen K.H., Granum B., Hjorthaug H.S., Undlien D., Lyle R. (2010). CD14 polymorphisms and serum CD14 levels through childhood: A role for gene methylation?. J. Allergy. Clin. Immunol..

[94] Frisdal E., Klerkx A.H., Le Goff W., Tanck M.W., Lagarde J.P., Jukema J.W., Kastelein J.J., Chapman M.J., Guerin M. (2005). Functional interaction between -629C/A, -971G/A and -1337C/T polymorphisms in the CETP gene is a major determinant of promoter activity and plasma CETP concentration in the REGRESS Study. Hum. Mol. Genet..

[95] Claussnitzer M., Dankel S.N., Kim K.H., Quon G., Meuleman W., Haugen C., Glunk V., Sousa I.S., Beaudry J.L., Puviindran V. (2015). FTO Obesity Variant Circuitry and Adipocyte Browning in Humans. N. Engl. J. Med..

[96] Bell C.G., Finer S., Lindgren C.M., Wilson G.A., Rakyan V.K., Teschendorff A.E., Akan P., Stupka E., Down T.A., Prokopenko I. (2010). Integrated genetic and epigenetic analysis identifies haplotype-specific methylation in the FTO type 2 diabetes and obesity susceptibility locus. PLoS ONE.

[97] Köttgen A., Glazer N.L., Dehghan A., Hwang S.J., Katz R., Li M., Yang Q., Gudnason V., Launer L.J., Harris T.B. (2009). Multiple loci associated with indices of renal function and chronic kidney disease. Nat. Genet..

[98] Prokop J.W., Yeo N.C., Ottmann C., Chhetri S.B., Florus K.L., Ross E.J., Sosonkina N., Link B.A., Freedman B.I., Coppola C.J. (2018). Characterization of Coding/Noncoding Variants for SHROOM3 in Patients with CKD. J. Am. Soc. Nephrol..

[99] Köttgen A., Pattaro C., Böger C.A., Fuchsberger C., Olden M., Glazer N.L., Parsa A., Gao X., Yang Q., Smith A.V. (2010). New loci associated with kidney function and chronic kidney disease. Nat. Genet..

[100] Ahluwalia T.S., Troelsen J.T., Balslev-Harder M., Bork-Jensen J., Thuesen B.H., Cerqueira C., Linneberg A., Grarup N., Pedersen O., Hansen T. (2017). Carriers of a VEGFA enhancer polymorphism selectively binding CHOP/DDIT3 are predisposed to increased circulating levels of thyroid-stimulating hormone. J. Med. Genet..

[101] Sun C.Y., Wu M.S., Lee C.C., Chen S.H., Lo K.C., Chen Y.H. (2018). A novel SNP in the 5’ regulatory region of organic anion transporter 1 is associated with chronic kidney disease. Sci. Rep..

[102] Gaulton K.J., Ferreira T., Lee Y., Raimondo A., Mägi R., Reschen M.E., Mahajan A., Locke A., Rayner N.W., Robertson N. (2015). Genetic fine mapping and genomic annotation defines causal mechanisms at type 2 diabetes susceptibility loci. Nat. Genet..

[103] Lyssenko V., Nagorny C.L., Erdos M.R., Wierup N., Jonsson A., Spégel P., Bugliani M., Saxena R., Fex M., Pulizzi N. (2009). Common variant in MTNR1B associated with increased risk of type 2 diabetes and impaired early insulin secretion. Nat. Genet..

[104] Fogarty M.P., Cannon M.E., Vadlamudi S., Gaulton K.J., Mohlke K.L. (2014). Identification of a regulatory variant that binds FOXA1 and FOXA2 at the CDC123/CAMK1D type 2 diabetes GWAS locus. PLoS Genet..

[105] Fromont C., Atzori A., Kaur D., Hashmi L., Greco G., Cabanillas A., Nguyen H.V., Jones D.H., Garzón M., Varela A. (2020). Discovery of Highly Selective Inhibitors of Calmodulin-Dependent Kinases That Restore Insulin Sensitivity in the Diet-Induced Obesity *in Vivo* Mouse Model. J. Med. Chem..

[106] Hiramoto M., Udagawa H., Ishibashi N., Takahashi E., Kaburagi Y., Miyazawa K., Funahashi N., Nammo T., Yasuda K. (2018). A type 2 diabetes-associated SNP in KCNQ1 (rs163184) modulates the binding activity of the locus for Sp3 and Lsd1/Kdm1a, potentially affecting CDKN1C expression. Int. J. Mol. Med..

[107] Elliott H.R., Shihab H.A., Lockett G.A., Holloway J.W., McRae A.F., Smith G.D., Ring S.M., Gaunt T.R., Relton C.L. (2017). Role of DNA Methylation in Type 2 Diabetes Etiology: Using Genotype as a Causal Anchor. Diabetes.

[108] Fogarty M.P., Panhuis T.M., Vadlamudi S., Buchkovich M.L., Mohlke K.L. (2013). Allele-specific transcriptional activity at type 2 diabetes-associated single nucleotide polymorphisms in regions of pancreatic islet open chromatin at the JAZF1 locus. Diabetes.

[109] Wesolowska-Andersen A., Zhuo Yu G., Nylander V., Abaitua F., Thurner M., Torres J.M., Mahajan A., Gloyn A.L., McCarthy M.I. (2020). Deep learning models predict regulatory variants in pancreatic islets and refine type 2 diabetes association signals. Elife.

[110] Adamska-Patruno E., Godzien J., Ciborowski M., Samczuk P., Bauer W., Siewko K., Gorska M., Barbas C., Kretowski A. (2019). The Type 2 Diabetes Susceptibility PROX1 Gene Variants Are Associated with Postprandial Plasma Metabolites Profile in Non-Diabetic Men. Nutrients.

[111] Cheng M., Huang X., Zhang M., Huang Q. (2020). Computational and functional analyses of T2D GWAS SNPs for transcription factor binding. Biochem. Biophys. Res. Commun..

[112] Claussnitzer M., Dankel S.N., Klocke B., Grallert H., Glunk V., Berulava T., Lee H., Oskolkov N., Fadista J., Ehlers K. (2014). Leveraging cross-species transcription factor binding site patterns: From diabetes risk loci to disease mechanisms. Cell.

[113] Piao X., Yahagi N., Takeuchi Y., Aita Y., Murayama Y., Sawada Y., Shikama A., Masuda Y., Nishi-Tatsumi M., Kubota M. (2018). A candidate functional SNP rs7074440 in TCF7L2 alters gene expression through C-FOS in hepatocytes. FEBS Lett..

[114] López Rodríguez M., Kaminska D., Lappalainen K., Pihlajamäki J., Kaikkonen M.U., Laakso M. (2017). Identification and characterization of a FOXA2-regulated transcriptional enhancer at a type 2 diabetes intronic locus that controls GCKR expression in liver cells. Genome. Med..

[115] Zhou Y., Oskolkov N., Shcherbina L., Ratti J., Kock K.H., Su J., Martin B., Oskolkova M.Z., Göransson O., Bacon J. (2016). HMGB1 binds to the rs7903146 locus in TCF7L2 in human pancreatic islets. Mol. Cell. Endocrinol..

[116] Susa S., Daimon M., Sakabe J., Sato H., Oizumi T., Karasawa S., Wada K., Jimbu Y., Kameda W., Emi M. (2008). A functional polymorphism of the TNF-alpha gene that is associated with type 2 DM. Biochem. Biophys. Res. Commun..

[117] Zheng X., Li J., Sheng J., Dai Y., Wang Y., Liu J., Xu Y. (2020). Haplotypes of the Mutated SIRT2 Promoter Contributing to Transcription Factor Binding and Type 2 Diabetes Susceptibility. Genes.

[118] Cui G., Tian M., Hu S., Wang Y., Wang D.W. (2020). Identifying functional non-coding variants in APOA5/A4/C3/A1 gene cluster associated with coronary heart disease. J. Mol. Cell. Cardiol..

[119] Roman T.S., Marvelle A.F., Fogarty M.P., Vadlamudi S., Gonzalez A.J., Buchkovich M.L., Huyghe J.R., Fuchsberger C., Jackson A.U., Wu Y. (2015). Multiple Hepatic Regulatory Variants at the GALNT2 GWAS Locus Associated with High-Density Lipoprotein Cholesterol. Am. J. Hum. Genet..

[120] Li X., Zhang Y., Zhang M., Wang Y. (2020). GALNT2 regulates ANGPTL3 cleavage in cells and in vivo of mice. Sci. Rep..

[121] Pfeiffer L., Wahl S., Pilling L.C., Reischl E., Sandling J.K., Kunze S., Holdt L.M., Kretschmer A., Schramm K., Adamski J. (2015). DNA methylation of lipid-related genes affects blood lipid levels. Circ. Cardiovasc. Genet..

[122] Lin S., Dai R., Lin R. (2017). A meta-analytic evaluation of cholesteryl ester transfer protein (CETP) C-629A polymorphism in association with coronary heart disease risk and lipid changes. Oncotarget.

[123] Dachet C., Poirier O., Cambien F., Chapman J., Rouis M. (2000). New functional promoter polymorphism, CETP/-629, in cholesteryl ester transfer protein (CETP) gene related to CETP mass and high density lipoprotein cholesterol levels: Role of Sp1/Sp3 in transcriptional regulation. Arterioscler. Thromb. Vasc. Biol..

[124] Musunuru K., Strong A., Frank-Kamenetsky M., Lee N.E., Ahfeldt T., Sachs K.V., Li X., Li H., Kuperwasser N., Ruda V.M. (2010). From noncoding variant to phenotype via SORT1 at the 1p13 cholesterol locus. Nature.

[125] Gurdasani D., Carstensen T., Fatumo S., Chen G., Franklin C.S., Prado-Martinez J., Bouman H., Abascal F., Haber M., Tachmazidou I. (2019). Uganda Genome Resource Enables Insights into Population History and Genomic Discovery in Africa. Cell.

[126] Fairoozy R.H., White J., Palmen J., Kalea A.Z., Humphries S.E. (2016). Identification of the Functional Variant(s) that Explain the Low-Density Lipoprotein Receptor (LDLR) GWAS SNP rs6511720 Association with Lower LDL-C and Risk of CHD. PLoS ONE.

[127] Yin Z., Zhao Y., Du H., Nie X., Li H., Fan J., He M., Dai B., Zhang X., Yuan S. (2020). A Key GWAS-Identified Genetic Variant Contributes to Hyperlipidemia by Upregulating miR-320a. iScience.

[128] Reschen M.E., Gaulton K.J., Lin D., Soilleux E.J., Morris A.J., Smyth S.S., O’Callaghan C.A. (2015). Lipid-induced epigenomic changes in human macrophages identify a coronary artery disease-associated variant that regulates PPAP2B Expression through Altered C/EBP-beta binding. PLoS Genet..

[129] Aminoff A., Ledmyr H., Thulin P., Lundell K., Nunez L., Strandhagen E., Murphy C., Lidberg U., Westerbacka J., Franco-Cereceda A. (2010). Allele-specific regulation of MTTP expression influences the risk of ischemic heart disease. J. Lipid. Res..

[130] Wen G., Wessel J., Zhou W., Ehret G.B., Rao F., Stridsberg M., Mahata S.K., Gent P.M., Das M., Cooper R.S. (2007). An ancestral variant of Secretogranin II confers regulation by PHOX2 transcription factors and association with hypertension. Hum. Mol. Genet..

[131] Zhang K., Chen Y., Wen G., Mahata M., Rao F., Fung M.M., Vaingankar S., Biswas N., Gayen J.R., Friese R.S. (2011). Catecholamine storage vesicles: Role of core protein genetic polymorphisms in hypertension. Curr. Hypertens. Rep..

[132] Subramanian L., Maghajothi S., Singh M., Kesh K., Kalyani A., Sharma S., Khullar M., Victor S.M., Swarnakar S., Asthana S. (2019). A Common Tag Nucleotide Variant in MMP7 Promoter Increases Risk for Hypertension via Enhanced Interactions With CREB (Cyclic AMP Response Element-Binding Protein) Transcription Factor. Hypertension.

[133] Mopidevi B., Kaw M.K., Sivankutty I., Jain S., Perla S.K., Kumar A. (2019). A polymorphism in intron I of the human angiotensinogen gene (hAGT) affects binding by HNF3 and hAGT expression and increases blood pressure in mice. J. Biol. Chem..

[134] Park S., Lu K.T., Liu X., Chatterjee T.K., Rudich S.M., Weintraub N.L., Kwitek A.E., Sigmund C.D. (2013). Allele-specific expression of angiotensinogen in human subcutaneous adipose tissue. Hypertension.

[135] Purkait P., Halder K., Thakur S., Ghosh Roy A., Raychaudhuri P., Bhattacharya S., Sarkar B.N., Naidu J.M. (2017). Association of angiotensinogen gene SNPs and haplotypes with risk of hypertension in eastern Indian population. Clin. Hypertens..

[136] Fjorder A.S., Rasmussen M.B., Mehrjouy M.M., Nazaryan-Petersen L., Hansen C., Bak M., Grarup N., Nørremølle A., Larsen L.A., Vestergaard H. (2019). Haploinsufficiency of ARHGAP42 is associated with hypertension. Eur. J. Hum. Genet..

[137] Bai X., Mangum K.D., Dee R.A., Stouffer G.A., Lee C.R., Oni-Orisan A., Patterson C., Schisler J.C., Viera A.J., Taylor J.M. (2017). Blood pressure-associated polymorphism controls ARHGAP42 expression via serum response factor DNA binding. J. Clin. Invest..

[138] Zhou J., Zhu Y., Cheng M., Dinesh D., Thorne T., Poh K.K., Liu D., Botros C., Tang Y.L., Reisdorph N. (2009). Regulation of vascular contractility and blood pressure by the E2F2 transcription factor. Circulation.

[139] Menzaghi C., Paroni G., De Bonis C., Soccio T., Marucci A., Bacci S., Trischitta V. (2006). The -318 C>G single-nucleotide polymorphism in GNAI2 gene promoter region impairs transcriptional activity through specific binding of Sp1 transcription factor and is associated with high blood pressure in Caucasians from Italy. J. Am. Soc. Nephrol..

[140] Wainford R.D., Carmichael C.Y., Pascale C.L., Kuwabara J.T. (2015). Gαi2-protein-mediated signal transduction: Central nervous system molecular mechanism countering the development of sodium-dependent hypertension. Hypertension.

[141] Singh J.A., Cleveland J.D. (2018). Gout and the risk of age-related macular degeneration in the elderly. PLoS ONE.

[142] Ketharnathan S., Leask M., Boocock J., Phipps-Green A.J., Antony J., O’Sullivan J.M., Merriman T.R., Horsfield J.A. (2018). A non-coding genetic variant maximally associated with serum urate levels is functionally linked to HNF4A-dependent PDZK1 expression. Hum. Mol. Genet..

[143] Chen M., Lu X., Lu C., Shen N., Jiang Y., Wu H. (2018). Soluble uric acid increases PDZK1 and ABCG2 expression in human intestinal cell lines via the TLR4-NLRP3 inflammasome and PI3K/Akt signaling pathway. Arthritis. Res. Ther..

[144] Ziablitsev D.S., Larin O.S. (2015). Influence of single nucleotide polymorphisms of vitamin D receptor-gene on the level of osteoassociated hormones linkage with postmenopausal osteoporosis. Fiziol. Zh..

[145] Arai H., Miyamoto K.I., Yoshida M., Yamamoto H., Taketani Y., Morita K., Kubota M., Yoshida S., Ikeda M., Watabe F. (2001). The polymorphism in the caudal-related homeodomain protein Cdx-2 binding element in the human vitamin D receptor gene. J. Bone. Miner. Res..

[146] Marozik P., Rudenka A., Kobets K., Rudenka E. (2021). Vitamin D Status, Bone Mineral Density, and VDR Gene Polymorphism in a Cohort of Belarusian Postmenopausal Women. Nutrients.

[147] Mann V., Hobson E.E., Li B., Stewart T.L., Grant S.F., Robins S.P., Aspden R.M., Ralston S.H. (2001). A COL1A1 Sp1 binding site polymorphism predisposes to osteoporotic fracture by affecting bone density and quality. J. Clin. Invest..

[148] Xie P., Liu B., Zhang L., Chen R., Yang B., Dong J., Rong L. (2015). Association of COL1A1 polymorphisms with osteoporosis: A meta-analysis of clinical studies. Int. J. Clin. Exp. Med..

[149] Zhu D.L., Chen X.F., Hu W.X., Dong S.S., Lu B.J., Rong Y., Chen Y.X., Chen H., Thynn H.N., Wang N.N. (2018). Multiple Functional Variants at 13q14 Risk Locus for Osteoporosis Regulate RANKL Expression Through Long-Range Super-Enhancer. J. Bone. Miner. Res..

[150] Lill C.M., Liu T., Norman K., Meyer A., Steinhagen-Thiessen E., Demuth I., Bertram L. (2016). Genetic Burden Analyses of Phenotypes Relevant to Aging in the Berlin Aging Study II (BASE-II). Gerontology.

[151] Huang Q., Whitington T., Gao P., Lindberg J.F., Yang Y., Sun J., Väisänen M.R., Szulkin R., Annala M., Yan J. (2014). A prostate cancer susceptibility allele at 6q22 increases RFX6 expression by modulating HOXB13 chromatin binding. Nat. Genet..

[152] Houlahan K.E., Shiah Y.J., Gusev A., Yuan J., Ahmed M., Shetty A., Ramanand S.G., Yao C.Q., Bell C., O’Connor E. (2019). Genome-wide germline correlates of the epigenetic landscape of prostate cancer. Nat. Med..

[153] Ramanand S.G., Chen Y., Yuan J., Daescu K., Lambros M.B., Houlahan K.E., Carreira S., Yuan W., Baek G., Sharp A. (2020). The landscape of RNA polymerase II-associated chromatin interactions in prostate cancer. J. Clin. Invest..

[154] Emami N.C., Cavazos T.B., Rashkin S.R., Graff R.E., Tai C.G., Mefford J.A., Kachuri L., Cario C.L., Wan E., Wong S. (2020). A large-scale association study detects novel rare variants, risk genes, functional elements, and polygenic architecture of prostate cancer susceptibility. Cancer Res..

[155] Whitington T., Gao P., Song W., Ross-Adams H., Lamb A.D., Yang Y., Svezia I., Klevebring D., Mills I.G., Karlsson R. (2016). Gene regulatory mechanisms underpinning prostate cancer susceptibility. Nat. Genet..

[156] Zhang P., Tillmans L.S., Thibodeau S.N., Wang L. (2019). Single-Nucleotide Polymorphisms Sequencing Identifies Candidate Functional Variants at Prostate Cancer Risk Loci. Genes.

[157] Wang X., Feng Z., Li J., Han Y., Su L., Wang F., Yang Y., Zhang Y. (2019). Functional Variant rs4442975 Modulating FOXA1 Binding Affinity Can Influence Bone Marrow Suppression during Neoadjuvant Chemotherapy for Luminal A Type Breast Cancer. Biomed. Res. Int..

[158] Patti G., Micieli G., Cimminiello C., Bolognese L. (2020). The Role of Clopidogrel in 2020: A Reappraisal. Cardiovasc. Ther..

[159] Depta J.P., Lenzini P.A., Lanfear D.E., Wang T.Y., Spertus J.A., Bach R.G., Cresci S. (2015). Clinical outcomes associated with proton pump inhibitor use among clopidogrel-treated patients within CYP2C19 genotype groups following acute myocardial infarction. Pharm. J..

[160] Frére C., Cuisset T., Gaborit B., Alessi M.C., Hulot J.S. (2009). The CYP2C19*17 allele is associated with better platelet response to clopidogrel in patients admitted for non-ST acute coronary syndrome. J. Thromb. Haemost..

[161] Frey U.H., Hauner H., Jöckel K.H., Manthey I., Brockmeyer N., Siffert W. (2008). A novel promoter polymorphism in the human gene GNAS affects binding of transcription factor upstream stimulatory factor 1, Galphas protein expression and body weight regulation. Pharm. Genom..

